# Soybean Nodule-Associated Non-Rhizobial Bacteria Inhibit Plant Pathogens and Induce Growth Promotion in Tomato

**DOI:** 10.3390/plants9111494

**Published:** 2020-11-05

**Authors:** Serkan Tokgöz, Dilip K. Lakshman, Mahmoud H. Ghozlan, Hasan Pinar, Daniel P. Roberts, Amitava Mitra

**Affiliations:** 1 Department of Plant Pathology, University of Nebraska-Lincoln, Lincoln, NE 68588, USA; srkntkgz@gmail.com (S.T.); mhgh_4@yahoo.com (M.H.G.); hpinarka@yahoo.com (H.P.); 2 Sustainable Agricultural Systems Laboratory, USDA-ARS, Beltsville, MD 20705, USA; dan.roberts@usda.gov

**Keywords:** root nodule, leguminous plants, rhizobia, non-rhizobial bacteria, soybean, disease control, growth promotion

## Abstract

The root nodules are a unique environment formed on legume roots through a highly specific symbiotic relationship between leguminous plants and nodule-inducing bacteria. Previously, Rhizobia were presumed to be the only group of bacteria residing within nodules. However, recent studies discovered diverse groups of bacteria within the legume nodules. In this report soybean nodule-associated bacteria were studied in an effort to identify beneficial bacteria for plant disease control and growth promotion. Analysis of surface-sterilized single nodules showed bacterial diversity of the nodule microbiome. Five hundred non-rhizobial colonies from 10 nodules, 50 colonies per nodule, were tested individually against the tomato wilt causing bacterial pathogen *Clavibacter michiganensis* subsp. *michiganensis* (Cmm) for inhibition of pathogen growth. From the initial screening, 54 isolates were selected based on significant growth inhibition of Cmm. These isolates were further tested in vitro on another bacterial pathogen *Pseudomonas syringae* pv. tomato (Pst) and two fungal pathogens *Rhizoctonia solani* and *Sclerotinia sclerotiorum*. Bacterial metabolites were extracted from 15 selected isolates with ethanol and tested against pathogen Cmm and Pst. These isolates were identified by using MALDI-TOF mass spectrometry and 16S rRNA gene sequencing. *Pseudomonas* spp. were the dominant soybean nodule-associated non-rhizobial bacterial group. Several isolates imparted significant protection against pathogens and/or plant growth promotion on tomato seedlings. The most promising nodule-associated bacterial isolate that suppressed both Cmm and Pst in vitro and Pst in tomato seedlings was identified as a *Proteus* species. Isolation and identification of beneficial nodule-associated bacteria established the foundation for further exploration of potential nodule-associated bacteria for plant protection and growth promotion.

## 1. Introduction

The rhizosphere is a narrow zone of soil encompassing plant roots and various organic compounds that have a direct influence on plant growth and performance [1]. It is also regarded as one of the most dynamic and complex interfaces due to numerous interactions within organisms residing in the rhizosphere. These interactions can be beneficial, including symbiotic relationships with beneficial microbes such as rhizobia, mycorrhiza, and plant growth-promoting rhizobacteria (PGPR), or they can be deleterious consisting of pathogenic microbes [2]. Many PGPR are known to interact with plants in the rhizosphere resulting in improved plant health, enhanced plant growth and crop yield [3,4,5]. 

The root nodule is an ecological niche formed in the legume roots through a highly specific symbiotic relationship between leguminous plants and nodule inducing bacteria. For many years, nitrogen fixing Rhizobia were assumed to be the only bacteria residing in the nodules of legumes [6]. However, it has now been shown that legume root nodules host a number of other microbial occupants [7]. These nodule-inhabitant microorganisms, apart from rhizobia, are now commonly referred to as non-rhizobia endophytes [8], nodule endophytes [9], or nodule-associated bacteria (NAB) [10]. Many studies have revealed that *Bacillus* is the most common genus detected as non-rhizobial endophytes in legume nodules, followed by the genus *Pseudomonas* [8]. These beneficial microorganisms are often antagonistic to plant pathogens via several mechanisms, such as antibiosis [11], hyperparasitism [12], production of lytic enzymes [13], and induction of plant innate immunity to reduce disease incidence and severity [14,15]. Species of the genus *Micromonospora* have been observed in several legume root nodules, which suppressed fungal diseases by inducing systemic resistance (ISR) in the host, and also acted as plant growth promotion bacteria when applied to soil [7]. Bacterial strains from the genera *Pseudomonas*, *Serratia*, *Bacillus*, and *Burkholderia* are also documented as potential inducers of systemic resistance (ISR) [16]. *Variovorax* is an intriguing genus found in legume root nodules producing broad spectrum hydrolytic enzymes like lipase, cellulase, and protease [17]. *Bacillus* spp. and *Pseudomonas* spp. are especially involved in inhibiting plant pathogens by producing antibiotics such as subtilin, bacilysin, chlorotetain, mycobacillin, 2,4 Diacetyl Phloroglucinol (DAPG), Phenazine-1-carboxylic acid (PCA), and Phenazine-1-carboxamide (PCN) [18]. 

Soybean (*Glycine max* L.) is a major economically important legume crop cultivated across the world. Soybean has a unique symbiotic relationship with Rhizobia, providing a remarkable nitrogen-fixing capability in root nodules [19]. Several Rhizobia species including *Bradyrhizobium japonicum, B. diazoefficiens, B. liaoningense, B. yuan-mingense, B. elkanii, B. huanghuaihaiense, B. daqingense, B. pachyrhizi, B. iriomotense, B. canariense, Sinorhizobium fredii,* and *S. sojae* have been isolated from soybean nodules in China, the center of origin of soybean [20]. In addition, a variety of nodule endophytes from the genera *Pantoea, Serratia, Acinetobacter, Bacillus, Agrobacterium*, and *Burkholderia* have also recently been identified in soybean nodules [20]. The awareness and possible roles of nodule endophytes may open new avenues for the biological control of plant diseases and the promotion of plant growth. 

Considering the importance of bacterial and fungal diseases of crop plants, a study was undertaken to identify soybean nodule endophytes for their disease suppression capabilities of economically important diseases. Soybean was chosen because it is the only legume widely cultivated in the state of Nebraska and the nodules were readily available from field grown crop plants. The main objectives of this work were to isolate and identify bacterial species from soybean nodules, screen antagonistic activities in vitro against bacterial and fungal pathogens, test for metabolites associated with disease control, and evaluate a few selected isolates for disease control and plant growth promotion potential in tomato seedlings. This study identified a few soybean nodule inhabiting bacterial species that imparted resistance against phytopathogens and growth enhancements in tomato. Isolation and identification of beneficial nodule-associated bacteria established the foundation for further exploration of potential nodule-associated bacteria for plant protection and growth promotion.

## 2. Results

### 2.1. Isolation of Soybean Nodule Bacteria

Nitrogen fixing nodules from a locally grown soybean field were used to isolate nodule-inhabiting bacteria. As the number of colonies was very high in the maceration extract from combined soybean root nodules, a single nodule was used for each isolation of bacterial colonies. Since Rhizobium colonies do not appear before 3–4 days of plating in the liquid Modified Arabinose Gluconate (MAG) medium, we colony-picked non-Rhizobial isolates on day 2 of incubation (Patrick Elia, USDA Rhizobia Bank, Beltsville, MD, personal communication). From each nodule, a total of 50 isolates were selected. A single colony from each isolate was further streaked on a plate for single colony purification. Hence, a pool of 500 pure culture of isolates were generated from 10 individual soybean nodules for subsequent in vitro and in vivo tests. The pool was also stored in glycerol stocks at −80 °C for future use.

### 2.2. In Vitro Antibacterial and Antifungal Bioassay

**Figure 1:**
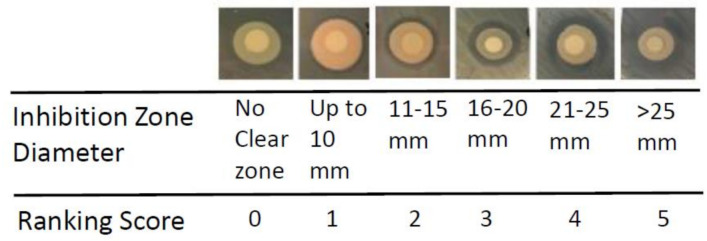
The ranking of in vitro antibacterial activity of nodule endophytes against bacterial pathogens. The inhibitory zones represent diameters in mm from the center of filter discs.

**Figure 2:**
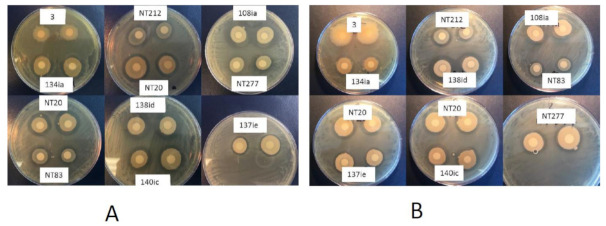
Antibacterial activity of select nodule endophytes against *Clavibacter michiganensis* subsp. *michiganensis* (**A**) and *Pseudomonas syringae* pv. tomato (**B**). Antagonism is indicated by the clear zone of lysis surrounding the filters containing a nodule bacterial endophyte.

All 500 isolates from 10 different soybean nodules were screened in vitro against an important bacterial plant pathogen *Clavibacter michiganensis* subsp. *michiganensis* (Cmm). The isolates were randomly divided into 10 groups with 50 isolates for each experiment. Ten experiments were conducted to cover all isolates for a single replication. A total of 1 mL of freshly grown Cmm culture was uniformly distributed on each YEP (10 g yeast extract, 10 g Bacto peptone, 5 g NaCl L^−1^, pH 7.0) agar plate with autoclaved cotton swabs, then sterilized 5 mm diameter filter paper discs were placed on the plates. A total of 25 μL of nodule isolates were spotted on separate filter discs and the plates were incubated at room temperature for 24 h. After 24 h, the antagonistic effect of a nodule-associated bacterial isolate was ranked by comparing clear zones around the filter discs using a 0 to 5 scale (0 means no effect, 5 means strong effect) (Figure 1). Of the 500 isolates, 54 isolates showed inhibition zones on plate assays ranked in scales from 1 to 5. Among these 54 isolates, 11 isolates coded as 3, 125ia, 125ib, 108ia, 108ic, 115ic, 138id, 138ia, NT76ie, NT76ia, and 113id showed the maximum inhibition zone with a score of 5 while 26 isolates exhibited smaller inhibition zone score of 4. The inhibition zone score was 3 for 11 isolates while three other isolates had a score of 2. The last three isolates had the narrowest clear zone around them and hence scored 1. The selected 54 isolates were also used in subsequent antagonistic tests against the bacterial pathogen *Pseudomonas syringae* pv. tomato (Pst). They all displayed in vitro antagonistic effect to the bacterial pathogen Pst, albeit to different degrees. Interestingly, 11 isolates which had the strongest antibacterial activities towards Cmm showed similar antagonistic behavior against Pst as well (Figure 2).

**Figure 3:**
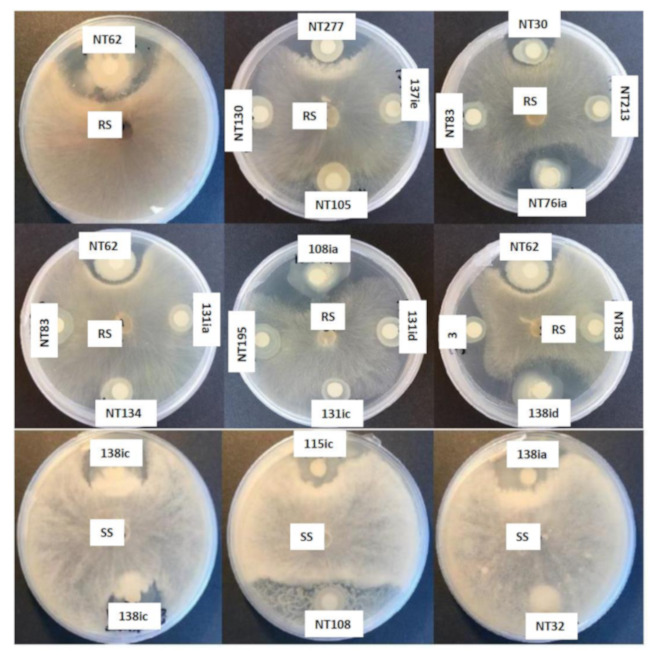
In vitro growth inhibitory activity of representative nodule endophytes against *Rhizoctonia solani* (RS) and *Sclerotinia sclerotiorum* (SS). Figure shows representative isolates.

Fifty four selected isolates were also screened for their potential antagonistic activities against two economically important fungal plant pathogens *Rhizoctonia solani* and *Sclerotinia sclerotiorum*. Among the 54 isolates, nine isolates exhibited growth inhibitory activities against *R. solani*. Especially, the isolate NT62 that showed a powerful growth inhibitory effect with a clear inhibition zone even after seven days of incubation at room temperature. On the other hand, five isolates showed weak inhibitory effects towards *S. sclerotiorum* (Figure 3).

### 2.3. In Vitro Metabolite Test

**Figure 4:**
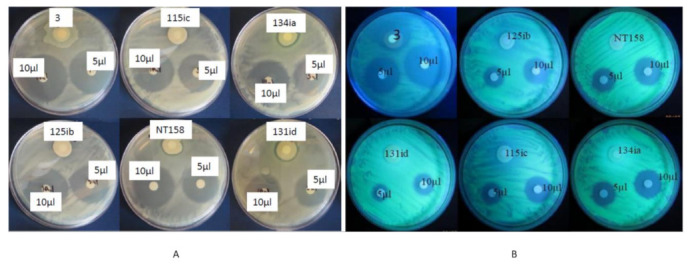
Antagonistic effects of metabolites extracted from a few selected nodule endophytes against *Clavibacter michiganensis* subsp. *michiganensis* (Cmm) (**A**) and GFP-tagged *Pseudomonas syringae* pv. *tomato* (Pst) (**B**, observed with a hand-held UV lamp) on solid plates. Antagonism is indicated by the clear zone of lysis of Cmm/Pst surrounding the filters containing extracts from selected nodule bacterial endophytes. Each plate contains one disk with 25 μL of bacterial inoculums (Cmm or Pst) and two discs containing 5 and 10 μL of metabolites isolated from one of the selected isolates. The plates were kept at room temperature for 24 h. Filter discs with 10 μL of water served as control.

Fifteen nodule-associated bacterial isolates, namely, 115ic, 3, NT158, NT21, NT76ia, 138id, 113id, NT134ia, 108ia, NT88, 131id, NT76ie, 108ic, 125ib, and 140ic, were selected based on previous screening against Cmm. The selection contained nine isolates scoring 5 and six isolates from other score groups. Of the metabolites extracted from these isolates, seven isolates—115ic, 134ia, 3, NT76ie, NT158, 125ib, and 131id—had inhibitory activities against a lawn of Cmm with substantial clear zones around the site where they were spotted on the YEP agar plates (Figure 4A). While the maximum inhibition zone was observed from the metabolite extracted from the nodule-associated bacterial isolate 3, which was later identified as *Proteus* sp., the metabolite extracted from the bacterial isolate 131id showed the minimum inhibition zone among the seven samples. The other five isolates exhibited similar inhibition zones, slightly narrower than that produced by the *Proteus* sp. These 15 metabolites were also used in a subsequent test against GFP-tagged Pst. However, their effectiveness was relatively low on Pst (about 50% lower than Cmm) except for the metabolite obtained from the *Proteus* sp., that showed similar inhibition against GFP-tagged Pst. (Figure 4B).

### 2.4. Identification of Bacterial Isolates

Matrix-assisted laser desorption/ionization-time of flight mass spectrometry (MALDI-TOF MS) is an emerging technology that is increasingly being used for bacterial identification. The system can identify a broad range of bacteria by matching unique fingerprints of abundant proteins from the bacterial cultures. A “direct smear” approach on two spots was used to analyze soybean nodule bacteria by MALDI-TOF MS and compared to the commercial database. Only five isolates were confirmed to species level IDs (score greater than or equal to 2.0), while the others could only be confirmed to the genus level, although best-match species were also suggested (Table 1). 

While MALDI-TOF MS provides a simple and cost-effective tool to rapidly identify unknown bacterial cultures, confirmed distinction beyond the genus level was not possible for most of the species evaluated in this study. As the success of this method is dependent on the availability of large robust data sets, future capability of MALDI-TOF MS is likely to improve with additions of plant-associated bacteria to the existing databases.

Further confirmation of identities of selected isolates from nodules was obtained by 16S rRNA sequencing. The UNVDC sequenced the 16S rRNA gene that allowed a better comparison and matching with the databases. However, bacterial isolate 131 ID showed matches below 95%, hence, species of this isolate could not be confirmed (Table 2).

### 2.5. Identification of Bacterial Endophytes in One Soybean Nodule

Total DNA extract from a single nodule was subjected to sequencing of 16S RNA genes at the UNVDC. The sequencing revealed the presence of both Rhizobial and non-Rhizobial bacterial species. The majority (88%) of the amplified sequences belonged to the various species of *Rhizobium* (data not shown). However, many other species were also identified as nodule inhabitant endophytes. *Pseudomonas* species representing 8% dominated the non-rhizobial group. Seven other genera including *Agrobacterium*, *Ochrobactrum*, *Burkholderia*, *Proteus*, *Enterobacter*, *Pantoea*, and *Acinatobacter* were also detected in the soybean nodule (Table 3). 

### 2.6. In Vivo Seedling Test against Pseudomonas syringae pv. Tomato (Pst)

**Figure 5:**
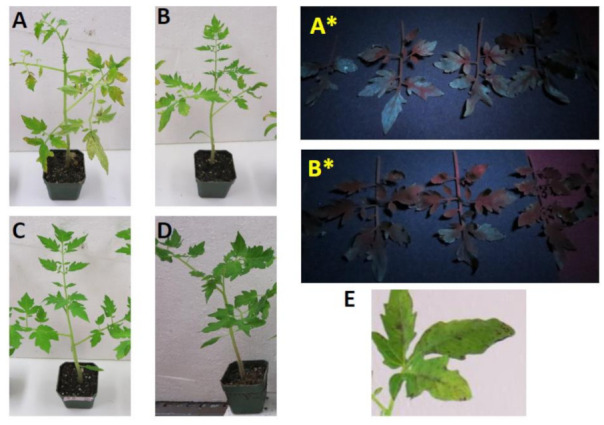
Suppression of bacterial speck disease on tomato plants following mix inoculation of *Pseudomonas syringae* pv. tomato with a nodule endophytic bacterium *Proteus* sp. (isolate 3) (**A**) Symptom development in tomato plants inoculated with GFP-tagged Pst alone, (A*) same leaves observed with a hand-held UV lamp. (**B**) Symptom development in tomato plants inoculated with mixed culture of Pst and nodule-associated isolate 3 (*Proteus* sp.), (B*) same leaves observed with a hand-held UV lamp. (**C**) Tomato plants inoculated with nodule-associated bacteria *Proteus* sp. alone. (**D**) Untreated control plants. (**E**) Magnified image of Pst inoculated leaf showing leaf-speck symptom.

Tomato plants exposed to GFP-tagged Pst culture suspension displayed leaf chlorosis in tomato five days after treatment, the typical characteristic symptoms of bacterial speck disease. However, there was a significant decrease in the chlorotic area on the leaves as well as the speck lesions when the tomato seedlings were treated with a mixed culture of GFP-tagged Pst and the nodule-associated bacteria *Proteus* sp. (Figure 5). The inoculation of tomato seedlings with mixed culture considerably reduced both disease severity and incidence in comparison to inoculation with the pathogen alone. Visual observation of inoculated leaves with a UV lamp indicated that *Proteus* sp. reduced the level of Pst in tomato leaves.

### 2.7. In Vivo Seedling Test against Clavibacter Michiganensis subsp. Michiganensis

Fifteen nodule-associated bacterial isolates were screened for their in vivo antagonistic potential against the bacterial pathogen Cmm on tomato plants in the greenhouse. Tomato seedlings inoculated with Cmm in greenhouse showed interveinal chlorosis of leaves and occasional unilateral leaf wilting. Comparisons of shoot height and shoot biomass were made between treated and untreated tomato plants to assess disease mitigating effects of nodule isolates. Shoot height was found significantly greater in plants treated with seven isolates, viz., 115ic, 138id, 113id, NT134ia, NT88, NT76ie, and 125ib in comparison to shoot height of the control plant. The maximum shoot height of 60.5 cm was observed in plants treated with isolate NT134ia, identified as *Pseudomonas putida*, followed by the isolates 125ib, 113id, and NT88 with shoot heights of 56.9, 56.9, and 56.6 cm, respectively (Table 4). While seven isolates showed a significant increase in shoot height, only three isolates, 125ib, 115ic, and 134ia exhibited a significant increase in shoot biomass, 72.9 g, 77.6 g, and 78 g, respectively, compared to 58.2 g shoot biomass of the control plant (Table 4).

**Figure 6:**
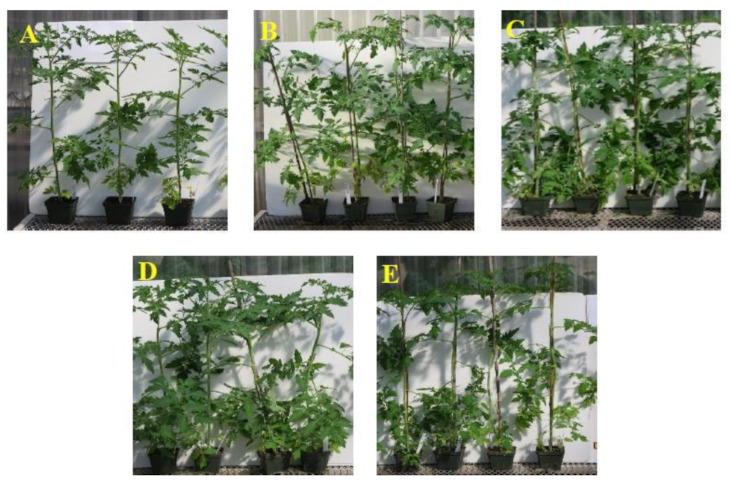
Effects of four selected endophytes on growth promotion of tomato plants grown in pasteurized potting mix. (**A**) Untreated control plants. (**B**) Treated with the isolate 140ic. (**C**) Treated with the isolate 108ia. (**D**) Treated with the isolate NT88. (**E**) Treated with the isolate NT134ia.

These 15 isolates were additionally evaluated for their growth promotion potential of tomato plants in the greenhouse. Tomato plants treated with the isolates NT134ia, 108ia, and NT88 had considerably higher shoot heights of 69.1 cm, 67.6 cm, and 66.8 cm, respectively, compared with the 60.2 cm shoot height of control plants (Table 5). Furthermore, tomato plants treated with these three isolates had significantly higher shoot biomass of 90.9 g, 89.5 g, and 95.3 g in comparison to the control plant of 76.1 g (Table 5, Figure 6). Interestingly, while the isolates 131id and 140ic significantly increased shoot biomass, they did not promote shoot height.

## 3. Discussion

Root nodule is a unique habitat established in the roots of leguminous plants via a highly specific symbiosis between legumes and nodule inducing bacteria. Unlike previous assumptions that Rhizobia were the only group of bacteria inhabiting legume root nodules, recent studies isolated and identified many bacterial species belonging to other bacterial genera from various legume root nodules. These bacteria are now commonly known as nodule endophytes, non-rhizobia endophytes or nodule-associated bacteria. Although the nodule-associated non-rhizobia bacteria now seem to be common within the legume nodules, their role or potential benefits are still unexplored. By understanding their roles and exploiting their potential benefits, these nodule-associated non-rhizobial bacteria may provide new opportunities to enhance agricultural production through their capability of plant growth promotion and biological control of plant diseases. In this study, nodule-associated bacteria from soybean nodules were isolated and identified. Their antagonistic activities were examined in vitro against a few selected economically important plant pathogenic fungi and bacteria. Additionally, the selected nodule endophytes were evaluated for their effectiveness for disease control and plant growth promotion in tomato plants.

Medium-sized pink nodules were collected from the roots of soybean plants that were at the R3-R4 growth stage. A single nodule generated a large number bacterial colonies, an indication that the nodule-associated bacteria are widely present in soybean nodules. A similar observation was made previously that isolated a large number of nodule endophytes from 150 soybean nodules [21]. For this study, a colony population of 500 was created from 10 soybean nodules with the selection of 50 isolates from each nodule. Nodule isolates were collected two days after incubation on solid plates to avoid most abundant and fast growing Rhizobial species. Hence, all slow growing bacterial species were missed in this study. Nevertheless, even two days of incubation generated a very large number of diverse bacterial species.

Although in vitro antimicrobial assay has been commonly used for the determination of biocontrol potential, it has been demonstrated that there is a poor correlation between in vitro antimicrobial activity and in vivo disease suppression [22]. Likewise, in our work, just two of the nine isolates showing the strongest antimicrobial activity in vitro exhibited effective disease suppression in greenhouse in planta tests. Nevertheless, in vitro antibiosis screening provides a simple and practical preliminary selection method to test a large number of samples in a short time [23]. In the present study, from the initial 500 colonies 54 isolates (11%) showed various degrees of in vitro antibacterial activity to Cmm. Only 11 isolates among the selected 54 isolates had a strong antagonistic effect on both bacterial pathogens Cmm and Pst. Intriguingly, isolate #3 identified as *Proteus* sp. had a unique swarming type of lysis compared to the other 53 isolates, displaying a clear full-plate inhibition zone around them against both the bacterial pathogens tested. The nodule isolates were less effective in antifungal activity against *S. sclerotiorum* and *R. solani*.

Initial screening involved testing all 500 isolates for in vitro antagonistic activity against Cmm on solid plates. These screenings were used to select 15 isolates for subsequent studies. The identity of the isolates was determined by MALDI-TOF mass spectrometry and 16S ribosomal RNA gene sequencing. In the past, *Bacillus* sp. were reported as the most prevalent non-rhizobial inhabitants in legume nodules [7]. The current study, however, found *Pseudomonas* as the most prevalent non-rhizobial genus among the selected 15 isolates. Detection from a single soybean nodule revealed that 88% of the nodule bacterial community is Rhizobia related species. *Pseudomonas* (8%) was also the dominant non-Rhizobia bacterial group within a single nodule endophytes. 

The production of metabolites such as antibiotics, lytic enzymes, and volatile compounds is one of the most important mechanisms associated with the biocontrol potential of an antagonist against plant pathogenic bacteria and fungi [24]. In this study, metabolites extracted from seven nodule endophyte isolates showed inhibitory effects against the plant pathogens Cmm and a GFP-tagged Pst as determined by the formation of inhibition zones on the plates. The metabolite extracted from the *Proteus* sp. had the widest inhibition zone on plates against both the pathogens. However, although the nodule endophyte *Proteus* sp. successfully decreased the disease incidence and severity of the GFP-tagged Pst in tomato seedlings when a mixed culture containing pathogen and antagonist inoculums was applied, this nodule endophyte was not able to suppress pathogenicity of Cmm in green house grown tomato plants. Only three nodule endophytes, 125ib, 115ic, and 134ia out of the seven isolates, produced metabolites displaying in vitro inhibitory activities towards both the bacterial pathogens and had a significant disease suppression performance against Cmm in the greenhouse experiments.

In planta assays of 15 selected nodule endophytes, the isolates 115ic, 125ib, and 134ia, identified as *Pseudomonas chlororaphis*, *Acinetobacter calcoaceticus*, and *Pseudomonas putida* respectively, had efficient disease suppression performance with increase in both shoot height and shoot weight of treated plants compared to untreated control plants grown in Cmm infested soil. The strains of *P. chlororaphis* were reported in previous studies as a potential biocontrol agent of tomato foot and root rot disease caused by *Fusarium oxysporum* and stem rot of canola caused by *S. sclerotiorum*, with the production of metabolites preventing mycelial growth and inducing plant defense system [25]. In addition to biocontrol activities, *P. chlororaphis* (isolate 108ia) also functioned as a plant growth promoter in the present study. While *Acinetobacter calcoaceticus* was reported as a potential plant growth promoter [26] and an inhibitor of fungal development [27] in previous works, this study showed its potential to control two bacterial diseases caused by Cmm and Pst. However, *A. calcoaceticus* did not exhibit significant growth promotion on tomato plants in the current study. The potential of *Pseudomonas putida* strains for plant growth promotion in several crops and biological control of both fungal and bacterial diseases by different mechanisms like induced systemic resistance have been demonstrated in several recent studies [28,29]. Similarly, the isolate identified as *P. putida* in our study exhibited both growth promotion and disease suppression. Additionally, the isolate NT88, identified only to the genus level as a *Pseudomonas* sp., increased shoot weight and height of treated tomato plants compared to untreated control plants grown in clean soil.

## 4. Materials and Methods

### 4.1. Isolation of Soybean Nodule Bacteria

#### 4.1.1. Collection and Surface Sterilization of Nodules

Short root segments of soybean cv. Williams 82 containing nodules were collected from R3–R4 stage plants grown at the University of Nebraska’s experimental field near Dead Man’s Run River. The roots were thoroughly washed under tap water to remove all dirt particles. Nodules were removed from the roots and were placed in a flask. The nodules were further washed several times with tap water until the wash water became clear. Nodules were surface sterilized by immersing them in 10% solution of commercial bleach (6% NaOCl) for 5 min followed by three washes with sterile distilled water. These nodules were then immersed in 70% EtOH for 30 s followed by three washes with sterile distilled water. Nodules were blotted dry on sterile paper towels and air dried for 5 min. Surface sterilized nodules were rolled over Yeast Extract Peptone (YEP) plates (100 mm X 15 mm) to test for contamination, only contamination-free nodules were used for bacterial isolation.

#### 4.1.2. Isolation of Bacteria from Surface-Sterilized Nodules

One surface-sterilized nodule was placed in an Eppendorf tube containing 300 μL of sterile distilled water. The nodule was gently squashed using a small spatula. The Eppendorf tube was then centrifuged at 6000 rpm for 30 s and the supernatant was transferred to a fresh Eppendorf tube. The volume was then adjusted to 1 mL with 0.5 X liquid Modified Arabinose Gluconate (MAG) media [30]. A dilution series was prepared from this extract and 100 μL aliquots were plated on solid MAG and incubated at room temperature for 2 days. The dilution series was needed to obtain well separated colonies on plates.

### 4.2. Plant Pathogenic Bacterial and Fungal Isolates 

Two bacterial plant pathogens, *Clavibacter michiganensis* subsp. *michiganensis* (Cmm), and *Pseudomonas syringae* pv. tomato (Pst), were used to screen antagonism of root nodule bacteria [31]. Similarly, antagonism of nodule bacteria was tested against two plant pathogenic fungi, *Sclerotinia sclerotiorum* (donated by J.F. Rollins, University of Florida) and *Rhizoctonia solani* AG4 isolate Rs23 [32]. A GFP-tagged Pst (gift from L. Zeng, University of Nebraska) was used in metabolite and tomato seedling assays.

### 4.3. Identification of Selected Bacterial Isolates 

The University of Nebraska Veterinary Diagnostic Center (UNVDC) core facility was used to determine the identity of fifteen selected bacterial isolates. The bacterial isolates were identified by two methods; first, the Matrix-assisted laser desorption/ionization-time of flight mass spectrometry (MALDI-TOF MS) [33] and subsequent confirmation using 16S based RNA gene sequencing. Single colony purified bacterial isolates were grown on fresh YEP plates and the cultures were provided to the core facility. Each colony was assayed three times. 

For MALDI-TOF MS, mass spectra from each culture were acquired and compared to a veterinary database using a software package (MALDI Biotyper Compass, Bruker Daltonics). Unknown soybean nodule bacterial cultures were identified to the genus or species level with scores of 2.0 or ≥2.3, respectively (out of a maximum of 3). The Same cultures were also used for bacterial identification by 16S ribosomal RNA gene sequencing. The entire 16S rRNA gene of each isolate was sequenced and BLASTN comparison against the NCBI GenBank databases was used for identification. Each bacterial isolate was sequenced bidirectionally. The UNVDC provided the MALDI scores and matches and 16S sequences and database matches of all 15 isolates.

### 4.4. Soybean Nodule Bacterial Community

To assess the diversity, a single soybean nodule was used to determine the bacterial community inside the nodule. A single pink nodule was surface sterilized as stated before and total DNA was extracted using PlantDNAzol (Invitrogen, Carlsbad, CA). The DNA samples were sent to the UNVDC core facility for species identification. The 16S amplicon sequences were matched with the NCBI database to identify bacterial species. Separate DNA extracts from three soybean nodules were used for sequencing, all three samples showed identical species composition.

### 4.5. In Vitro Screening of Potential Antagonistic Activity of Soybean Nodule-Associated Bacteria against Bacterial and Fungal Plant Pathogens

#### 4.5.1. Preparation of Bacterial Pathogen Inoculum

A 150 mL broth was prepared and autoclaved in a 250 mL Erlenmeyer flask. Autoclaved YEP broth was inoculated separately with the plant pathogens Cmm and Pst, and then incubated for 24 h at 25 °C on a rotary shaker at 180 rpm and for 24 h at 28 °C on a rotary shaker at 180 rpm, respectively. Fifty ml of overnight grown cultures were transferred into 50 mL centrifuge tubes. The tubes were centrifuged at 5000 rpm at 10 °C for 10 min to pellet the bacterial cells. The supernatant was discarded and the bacterial cell pellet was diluted with autoclaved YEP broth to obtain 1 × 10^8^ CFU mL^−1^.

#### 4.5.2. Preparation of Antagonist Inoculums

A 10 mL autoclaved YEP broth was transferred into 12 mL BD disposable tubes, inoculated with potential antagonistic nodule-associated bacterial isolates and incubated for 24 h at 25 °C on a rotary shaker at 180 rpm. The overnight grown cultures were processed as described for the pathogen inoculum.

#### 4.5.3. In Vitro Antifungal Bioassay

Two important soil pathogens *R. solani* (AG4) and *S. sclerotiorum* were chosen as fungal pathogens. Fifty-four nodule isolated bacteria determined as antagonist to bacterial pathogen Cmm in previously conducted antibacterial bioassays were tested against these two fungal pathogens. PDA (Potato Dextrose Agar, Difco Laboratories, Detroit, MI, USA) medium was prepared and autoclaved. Fungal pathogens were grown on PDA medium for 7 days. Five mm diameter mycelial plugs were removed from 7 days old actively growing fungal pathogen cultures and placed at the center of new PDA plates. Sterilized 5 mm diameter filter paper discs were placed around these PDA plugs, approximately 20 mm apart. a Total of μL of antagonist inoculum was spotted on these filter discs and then the PDA plates were incubated at room temperature for 4 days. Three PDA plates were used in each experiment and the experiment was repeated two more times.

### 4.6. In Vitro Screening of Metabolites 

#### 4.6.1. Extraction of Metabolites

Secondary metabolites were extracted from the selected 15 nodule-associated bacteria. Fresh cultures were grown on YEP agar plates for 24 h at room temperature. A total of 10 mL of 100% ethanol was added to freshly grown plates and incubated for 30 min at room temperature with gentle agitation. After 30 min, liquids were transferred into centrifuge tubes and centrifuged at 5000 rpm at 4 °C for 10 min. The supernatant was decanted into autoclaved glass petri plates. The plates were placed in a nitrogen desiccator and kept in a cold room at 4 °C until the liquid was completely evaporated. Dried metabolites left on petri plates were dissolved in 1 mL autoclaved double distilled water and transferred to 2 mL Eppendorf tubes.

#### 4.6.2. In Vitro Metabolite Test

A total of 1 mL aliquot from pathogen cultures was homogenously spread on each YEP agar plates with autoclaved cotton swaps and then three sterilized 5 mm diameter filter paper discs were equidistantly placed on these YEP agar plates. Each plate contained one disc with 25 μL of bacterial inoculum from one of the 15 nodule isolates and two discs containing 5 and 10 μL of metabolites isolated from the same isolate. The plates were incubated at room temperature for 24 h. Filter discs with 10 μL of water served as control. Visual observations were taken after 24 h. Three plates were used for each metabolite extract and the experiment was repeated three times.

### 4.7. In Vivo Seedling Test

The liquid cultures of pathogen (Cmm, Pst) and antagonist (nodule isolates) were mixed together in a 1:1 ratio to maintain a consistent inoculum dose during the experiment. Tomato seedlings (cv. Roma) were grown to two true leaf stage, which took approximately 20 days after seed germination. The aerial parts of 20-day-old tomato seedlings were dipped in pathogen inoculum, antagonist inoculum, or mixed inoculum for two minutes. Control plants were dipped in regular YEP broth. The inoculated seedlings were incubated for five days in plastic chambers (22 1/2″ L × 16″ W × 12 3/4″ H) at 24 °C under 16 h light: 8 h dark photoperiod. In all experiments, at least five seedlings were used for each bacterial isolate, and the experiments were repeated a minimum of three times.

#### In Planta Test

A day before the transplantation, 50 mL of pathogen inoculum (2 × 10^8^ CFU mL^−1^) and 300 mL sterile distilled water were mixed with 2.5 kg autoclaved soil (standard greenhouse mix; 5 gallons peat, 3 gallons soil, 2.5 gallons sand, 2.5 gallons vermiculite) to prepare infested soil and incubated at 25 °C overnight. Fifteen nodule-associated bacterial isolates viz., 115ic, 3, NT158, NT21, NT76ia, 138id, 113id, NT134ia, 108ia, NT88, 131id, NT76ie, 108ic, 125ib, and 140ic were prepared as before (1 × 10^8^ CFU mL^−1^ in 0.9% saline water containing 2% gum arabic) and used as biocontrol agents (BCA). 

Tomato seedlings (cv. Roma) at the two true leaf stage were used for in planta experiments. For the treatment, the root zone soil of 20-day-old tomato seedlings was washed off with distilled water and then the roots were dipped in respective BCA solutions for ten minutes. Treated tomato seedlings were transplanted in Cmm infested potting mix. For the control groups, the roots of tomato seedlings were dipped in 0.9% saline water containing 2% Gum arabic and then they were transplanted into the sterile potting mix or in Cmm infested potting mix. In addition, the roots of tomato seedlings were dipped in the respective BCA solutions for five minutes and transplanted into the pasteurized potting mix to determine their growth promotion potential on tomato plants. Five seedlings were used for each treatment and the entire experiment was repeated three times. Following transplantation, the seedling of specific treatments were drenched with 5 mL of the respective BCA solutions. Water was used for uninfected control plants. The plantlets were then incubated for ten days in plastic boxes as mentioned earlier at 24 °C under 16 h light: 8 h dark photoperiod with high humidity (over 90% RH) and then grown in greenhouse for 20 days under 16 h light: 8 h dark photoperiod with day and night temperatures of 25 °C and 20 °C, respectively.

### 4.8. Statistical Analysis 

Software R 3.3 was used to analyze differences among treatment means [34]. Fisher’s least significant difference test (LSD) was used for pairwise comparisons using statistical probability *p* ≤ 0.05.

## 5. Conclusions

In summary, this investigation confirms recent reports that soybean nodules harbor a large number of nodule endophytes from diverse genera in addition to traditional resident Rhizobia. This study also demonstrated for the first time that soybean nodule endophytes have great potentials for plant health management as well as growth promotion. Further studies are needed to fine-tune their efficient utilization and incorporation into crop production and protection systems in sustainable agriculture.

## Figures and Tables

**Table 1:** Bacterial identification based on Matrix-assisted laser desorption/ionization-time of flight mass spectrometry. Two independent colonies were assayed from each isolates. Same colony assayed three time generated identical data.

Sample ID	MALDI TOF MS Based ID	Score
108 ia	*Pseudomonas chlororaphis*	1.91
113 id	*Pseudomonas cepacia*	1.97
115 ic	*Pseudomonas chlororaphis*	1.93
134 ia	*Pseudomonas putida*	2.32
138 id	*Pseudomonas stutzeri*	1.96
NT 21	*Enterobacter cloacae*	2.33
NT 76 ia	*Pseudomonas acidovorans*	2.36
108 ic	*Pseudomonas chlororaphis*	2.12
140 ic	*Pseudomonas fluorescens*	1.93
NT 76 ie	*Pseudomonas brassicacearum*	1.89
125 ib	*Acinetobacter calcoaceticus*	1.84
3	*Proteus hauseri*	1.96
NT 158	*Enterobacter cloacae*	2.40
131 id	*Pseudomonas putida group*	1.98
NT 88	*Ochrobactrum* species	1.97

**Table 2:** Identification of individual nodule-associated bacterial isolates based on 16S rRNA gene amplicon.

Sample ID	16S Based ID	Closest Genbank Match (>99%)
108 ia	*Pseudomonas chlororaphis*	NBAT01000000
113 id	*Pseudomonas cepacia*	CP011301
115 ic	*Pseudomonas chlororaphis*	NBAT01000000
134 ia	*Pseudomonas putida*	AP013070
138 id	*Pseudomonas stutzeri*	CP003071
NT 21	*Enterobacter cloacae*	EU733519
NT 76 ia	*Pseudomonas acidovorans*	CP00884
108 ic	*Pseudomonas chlororaphis*	NBAT01000000
140 ic	*Pseudomonas fluorescens*	CP005975
NT 76 ie	*Pseudomonas brassicacearum*	NHAS01000000
125 ib	*Acinetobacter calcoaceticus*	AB859067
3	*Proteus hauseri*	CO5028
NT 158	*Enterobacter cloacae*	KJ668861
131 id	*Pseudomonas species*	
NT 88	*Ochrobactrum anthropi*	AB778290

**Table 3:** Non-rhizobial bacterial genera found in a single soybean nodule. Separate DNA extracts made from three soybean nodules were used for individual 16S sequencing, three individual nodules showed identical species composition.

Genus	Family	Order	Class
*Agrobacterium* spp.	Rhizobiaceae	Rhizobiales	Alphaproteobacteria
*Ochrobactrum* sp.	Brucellaceae	Rhizobiales	Alphaproteobacteria
*Burkholderia* spp.	Burkholderiaceae	Burkholderiales	Betaproteobacteria
*Pseudomonas* spp.	Pseudomonadaceae	Pseudomonadales	Gammaproteobacteria
*Proteus* spp.	Enterobacteriaceae	Enterobacteriales	Gammaproteobacteria
*Enterobacter* spp.	Enterobacteriaceae	Enterobacteriales	Gammaproteobacteria
*Pantoea* spp.	Enterobacteriaceae	Enterobacteriales	Gammaproteobacteria
*Acinatobacter* spp.	Moraxellaceae	Pseudomonadales	Gammaproteobacteria

**Table 4:** Evaluation of 15 nodule-associated bacteria for their antagonistic potential against *Clavibacter michiganensis* subsp. *michiganensis* in tomato plants in pathogen-infested soil under greenhouse conditions. A minimum of five seedlings were used for each treatment and the entire experiment was repeated three times. Control treatment comprised inoculation only with Cmm.

Treatments	Shoot Height (cm)	Shoot Biomass (g)
Control	50 ± 8.8	58.2 ± 17.2
115ic	55.4 ± 7.9 *	77.6 ± 29.3 **
3	54.1 ± 5.1	67.1 ± 37.2
NT158	53.8 ± 11.8	71.3 ± 30.4
NT21	52.5 ± 7.8	72.3 ± 29.6
NT76ia	52.1 ± 12.3	61.1 ± 25.8
138id	55.4 ± 12.4 *	64.7 ± 29.6
113id	56.9 ± 9 **	59.1 ± 23.8
NT134ia	60.5 ± 5.4 ***	78.0 ± 19.7 **
108ia	53.8 ± 7.8	58.0 ± 22.1
NT88	56.6 ± 9 **	59.1 ± 21.9
131id	53.1 ± 6.7	66.8 ± 20.4
NT76ie	56.1 ± 4.5 *	61.4 ± 23.7
108ic	51.1 ± 4.9	57.9 ± 22.7
125ib	56.9 ± 8.7 **	72.9 ± 14.5 *
140ic	52.6 ± 8.7	58.5 ± 23.1

* Statistically significant at 0.05 (*p* values). ** Statistically significant at 0.01 (*p* values). *** Statistically significant at 0.001 (*p* values).

**Table 5:** Evaluation of the fifteen nodule-associated bacteria for their growth promotion potential in tomato plants under greenhouse conditions. A minimum of five seedlings were used for each treatment and the entire experiment was repeated three times.

Treatments	Shoot Height (cm)	Shoot Biomass (g)
Control	60.2 ± 5.7	76.1 ± 20.4
115ic	59.7 ± 11.1	81.1 ± 22.2
3	55.1 ± 11.2	70.7 ± 19.7
NT158	58.7 ± 13.8	69.3 ± 16.5
NT21	55.6 ± 9.2	74.6 ± 22.7
NT76ia	59.4 ± 9.9	69.2 ± 24.4
138id	64.3 ± 4.3	85.1 ± 18.8
113id	61.7 ± 5.5	84.0 ± 20.8
NT134ia	69.1 ± 7.9 ***	90.9 ± 17.2 *
108ia	67.6 ± 4.8 **	89.5 ± 13.2 *
NT88	66.8 ± 3.3 *	95.3 ± 12.2 **
131id	59.4 ± 9.2	88.5 ± 29.2 *
NT76ie	59.7 ± 6.1	78.8 ± 24.5
108ic	60.5 ± 8.4	79.6 ± 19.5
125ib	59.2 ± 9.7	77.5 ± 17.8
140ic	60.2 ± 11.1	89.5 ± 26.7 *

* Statistically significant at 0.05 (*p* values). ** Statistically significant at 0.01 (*p* values). *** Statistically significant at 0.001 (*p* values).
